# A Systematic Review Exploring the Cardiovascular and Renal Effects of Empagliflozin in Patients With Heart Failure With Reduced Ejection Fraction

**DOI:** 10.7759/cureus.29896

**Published:** 2022-10-04

**Authors:** Sreekartthik Athiyaman, Bhawna Randhi, Sai Dheeraj Gutlapalli, Jingxiong Pu, Maheen F Zaidi, Maithily Patel, Lakshmi Malvika Atluri, Natalie A Gonzalez, Navya Sakhamuri, Sathish Venugopal

**Affiliations:** 1 Internal Medicine, California Institute of Behavioral Neurosciences & Psychology, Fairfield, USA; 2 Medicine, NRI Medical College, Chinakakani, IND; 3 Medicine, California Institute of Behavioral Neurosciences & Psychology, Fairfield, USA; 4 Psychiatry and Behavioral Sciences, California Institute of Behavioral Neurosciences & Psychology, Fairfield, USA; 5 Medicine, Aga Khan University, Karachi, PAK; 6 Research, California Institute of Behavioral Neurosciences & Psychology, Fairfield, USA; 7 Family Medicine, California Institute of Behavioral Neurosciences & Psychology, Fairfield, USA; 8 General Surgery, California Institute of Behavioral Neurosciences & Psychology, Fairfield, USA; 9 Surgery, Dr. Pinnamaneni Siddhartha Institute of Medical Science, Gannavaram, IND; 10 Pediatrics, Medical University of Graz, Graz, AUT; 11 Pediatrics, California Institute of Behavioral Neurosciences & Psychology, Fairfield, USA; 12 Neurology, California Institute of Behavioral Neurosciences & Psychology, Fairfield, USA

**Keywords:** sglt2 inhibitor, systolic heart failure, renal effects, cardiovascular effects, empagliflozin, heart failure with reduced ejection fraction

## Abstract

The major cause of death in the United States is heart disease. The global burden of illness and mortality from heart failure is substantial. Despite recent innovations in the treatment of heart failure, the prognosis is still poor. To identify, evaluate, and summarize the findings of all relevant studies of a drug that is equally efficacious but rather cost-effective, empagliflozin compared to the other sodium-glucose cotransporter-2 (SGLT2) inhibitors was studied. It is licensed by the Food and Drug Administration (FDA), acts by preventing the reabsorption of glucose from the kidney, and exhibits promising advantages in heart failure.

We systematically explored PubMed, PubMed Central (PMC), and Medical Literature Analysis and Retrieval System Online (MEDLINE) for randomized controlled trials (RCTs) and observational studies related to cardiovascular and renal outcomes of empagliflozin in patients with heart failure with reduced ejection fraction (HFrEF). After performing scoping search and search strategy, studies were screened for quality assessment using the Cochrane risk of bias assessment tool. We screened 60 articles by titles, abstract, and exclusion and inclusion criteria, after which eight final randomized controlled trials (RCTs) with 18,659 participants treated with empagliflozin and placebo were used for the systematic review. This systematic review's objective is to investigate and explore the full range of empagliflozin's effects and advantages on cardiac structure, function, and hemodynamics and renal function in patients with heart failure with reduced ejection fraction (EF) in order to better understand the drug's effects and related mechanisms.

## Introduction and background

The prevalence of heart failure in the United States from the years 2013 to 2016 was 6.2 million [1]. The approximate prevalence worldwide includes 26 million people, which also contributes to the financial burden worldwide [2]. The prevalence is higher in older age, around 4.3% in the age group 65-70 years in 2012, which is predicted to steadily rise to 8.5% in 2030 [3]. Therefore, it is usually considered a geriatric cardiovascular syndrome with a high burden of morbidity and mortality [3]. There are multiple etiologies for heart failure; the most common etiology could vary based on the region depending on the strength of implemented primary prevention measures [4]. It was historically attributed to coronary artery disease and myocardial infarction (MI) [4]. Over time, diabetes mellitus has been considered an important etiology besides coronary artery disease [4]. The prevention of heart failure is vital for those at risk. Many attempts have been made to identify this population. The American Heart Association classified this group as heart failure stage A, in order to facilitate early recognition and implementation of preventive measures [5]. It is important to decrease overall hospitalization rates and reduce the overall burden of this disease [5].

The latest medical advances guide us to effectively manage patients with heart failure with reduced ejection fraction (HFrEF) [6]. The treatment includes the following: renin-angiotensin inhibitors (angiotensin receptor/neprilysin inhibitors), angiotensin-converting enzyme inhibitors (ACEI), or angiotensin receptor blockers (ARB), beta-blockers, and mineralocorticoid receptor antagonists [6]. Sodium-glucose cotransporter-2 (SGLT2) inhibitor, an antihyperglycemic medication, reduces the risk of hospitalization for heart failure patients and cardiovascular death, which makes it unique compared to other medications in this category [7]. Among the available SGLT2 inhibitors in the market, empagliflozin had a low cost needed to treat diabetic patients for primary prevention compared to other agents [8].

In our systematic review, we evaluated the effects of empagliflozin in patients with heart failure with reduced ejection fraction (HFrEF) across different subgroups of associated diseases and risk factors to study the wide spectrum of benefits of the drug in these patients.

## Review

Methods

The Preferred Reporting Items for Systematic Reviews and Meta-Analyses (PRISMA) 2020 guidelines were employed for performing this systematic review [9].

Search Source and Search Strategy

We systematically searched for the relevant articles indexed in PubMed, PubMed Central (PMC), and Medical Literature Analysis and Retrieval System Online (MEDLINE). We began by exploring the databases using two search keywords. We combined these two keywords with the Boolean "AND." For the keyword "empagliflozin," we used the concept identification words sodium-glucose cotransporter-2 (SGLT2) inhibitor and empagliflozin; these words were combined by the Boolean "OR," and a Medical Subject Heading (MeSH) search strategy was formulated. Similarly, a MeSH search strategy for the second keyword "heart failure" was formulated, and both the search strategies were combined with the Boolean "AND," which was entered in PubMed. The following MeSH strategies were employed: ("empagliflozin" {Supplementary Concept}) AND ("Sodium-Glucose Transporter 2 Inhibitors/administration and dosage" {Majr} OR "Sodium-Glucose Transporter 2 Inhibitors/adverse effects" {Majr} OR "Sodium-Glucose Transporter 2 Inhibitors/therapeutic use" {Majr} OR "Sodium-Glucose Transporter 2 Inhibitors/toxicity" {Majr}) AND ("Heart Failure/drug therapy" {Mesh} OR "Heart Failure/prevention and control" {Mesh} OR "Heart Failure/therapy" {Mesh}).

The titles, abstracts, and subject headers were checked for relevance following a thorough analysis of all articles and references to verify that no publications that might be of interest were missed. The primary and secondary outcomes were determined, and the corresponding authors extracted the data. A consensus was reached to resolve any disagreements in the data extraction process.

Eligibility Criteria

The publications that are pertinent to the research issue and have been published in English throughout the previous six years, from 2017 to 2018, were included. Our study concentrated on adults (18 years and older). Prior to 2017, papers on pediatric populations and animal species that were unpublished, irrelevant, or considered "gray literature" were not included.

Risk of Bias Assessment

We used the Cochrane bias assessment tool to assess the quality of the randomized controlled trials included in our review article. Only those articles that satisfied >60% of the appraisal parameters were considered in the systematic review.

Results

Study Selection

**Figure 1:**
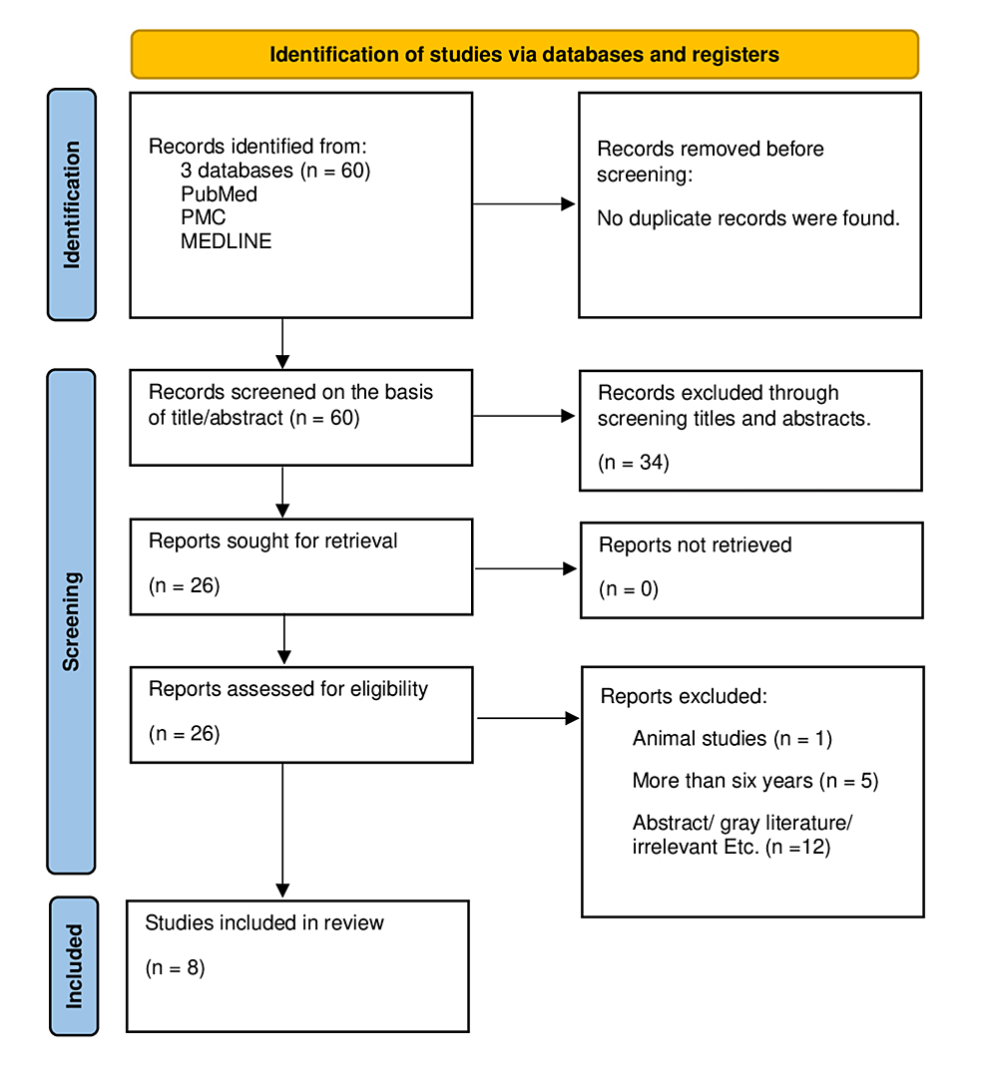
Preferred Reporting Items for Systematic Reviews and Meta-Analyses (PRISMA) flow diagram PMC: PubMed Central; MEDLINE: Medical Literature Analysis and Retrieval System Online

Our initial search employing the Medical Subject Heading (MeSH) strategy and keywords yielded sixty articles from the PubMed, PMC, and Medical Literature Analysis and Retrieval System Online (MEDLINE) databases. After performing scoping search through titles and abstracts, 34 articles were removed, and 26 articles remained. The 26 articles were reviewed for eligibility, and those that did not fit the requirements were removed, leaving eight articles. These eight articles were further subjected to quality appraisal since they included specific information relating to the research topic after reading the entire article. Since they all met more than 60% of the evaluation criteria and were randomized controlled trials, they were all employed. These articles mentioned the cardiac and renal effects of empagliflozin in patients with heart failure with reduced ejection fraction (EF). The literature screening process of the selection of eligible studies was performed employing the Preferred Reporting Items for Systematic Reviews and Meta-Analyses (PRISMA) guidelines as shown in the flow diagram in Figure 1 [9].

Quality Appraisal Studies

This systematic review includes eight randomized controlled trials, and the quality of RCTs was assessed using the Cochrane bias assessment tool. The findings are summarized in Table 1.

**Table 1:** Quality appraisal of randomized controlled trials using the Cochrane bias assessment tool + indicates a low risk of bias, and - indicates a high risk of bias

Author and year of publication	Random sequence generation	Allocation concealment	Finding of participants and personnel	Blinding of outcome assessment	Incomplete outcome data	Selective reporting	Other bias
Anker et al., 2021 [10]	+	+	+	+	-	-	-
Böhm et al., 2021 [11]	+	+	+	+	-	-	Selection bias
Fitchett et al., 2019 [12]	+	+	+	+	-	-	Post hoc nature
Lee et al., 2021 [13]	+	+	+	+	-	-	Selection bias
Omar et al., 2020 [14]	+	+	+	+	-	-	Observer bias
Omar et al., 2021 [15]	+	+	+	+	-	-	Post hoc nature
Santos-Gallego et al., 2021 [16]	+	+	+	+	-	-	Dropouts
Zannad et al., 2020 [17]	+	+	+	+	-	-	-

Characteristics of the Retrieved Studies

Our systematic review included eight randomized controlled trials with a total of 18,659 participants with heart failure with reduced ejection fraction (HFrEF). They are distributed under two sections: section 1 included four RCTs depicting the cardiac and renal effects of empagliflozin compared with a placebo in patients with HFrEF [10-12,17], whereas section 2 included the RCTs depicting the effects of empagliflozin on cardiac structure/function and hemodynamics [13-16].

The cardiac and renal beneficial outcomes of empagliflozin in patients with HFrEF include a significant reduction of cardiovascular death, hospitalization for heart failure, and a slowed rate of decline in estimated glomerular filtration rate (eGFR) and were observed in patients with type 2 diabetes mellitus (T2DM), and the baseline glycosylated hemoglobin (HbA1c) level did not influence the effect of the drug on the outcomes [10]. These findings were similar across the systolic blood pressure (SBP) spectrum as well; the SBP had no influence on the beneficial outcomes of the drug even at the lower end of the spectrum [11]. The beneficial cardiac and renal outcomes of the drug were similar across a broad range of baseline kidney function and in patients with chronic kidney disease (CKD) including patients with eGFR as low as 20 ml/minute/1.73 m^2^ [17]. Another subgroup of patients with higher cardiovascular risk, patients with and without prior myocardial infarction (MI) and/or stroke, also shared the same results including a relative reduction in three-point major adverse cardiac event (MACE) and all-cause mortality without impacting beneficial outcomes of the drug empagliflozin [12]. These were the interpreted beneficial outcomes from the RCTs of section 1.

The beneficial outcomes on cardiac structure/function and hemodynamics include a significant reduction of left ventricular (LV) end-systolic volume index (LVESVI), left ventricular end-diastolic volume index (LVEDVI), left atrial volume index (LAVI), left ventricular mass index, left ventricular sphericity, N-terminal pro-B-type natriuretic peptide (NT-pro-BNP), and pulmonary capillary wedge pressure (PCWP) and the improvement of left ventricular ejection fraction (LVEF), peak oxygen consumption, oxygen uptake efficiency slope, six-minute walk test, and quality of life [13-16]. These were the interpreted beneficial outcomes from the RCTs of section 2. The study characteristics of the eight randomized controlled trials depicting the effects of empagliflozin are outlined in Table 2.

**Table 2:** Study characteristics of the eight randomized controlled trials (RCTs) depicting the effects of empagliflozin SGLT2: sodium-glucose cotransporter-2; HFrEF: heart failure with reduced ejection fraction; eGFR: estimated glomerular filtration rate; MACE: major adverse cardiovascular event; T2DM: type 2 diabetes mellitus; ASCVD: atherosclerotic cardiovascular disease; MI: myocardial infarction; LVESVI: left ventricular end-systolic volume index; LVESV: left ventricular end-systolic volume; LVEDVI: left ventricular end-diastolic volume index; LVEDV: left ventricular end-diastolic volume; LAVI: left atrial volume index; LVEF: left ventricular ejection fraction; PCWP: pulmonary capillary wedge pressure; CI: cardiac index; CKD: chronic kidney disease

Author	Year	Participants	Type of study	Purpose	Results/conclusion
Anker et al. [10]	2020	3730	RCT	To investigate if glycemic status influences the magnitude of SGLT2 inhibitor's effects on both heart failure (HF) and renal events	Glycemic status had no significant interaction. Empagliflozin significantly improved cardiovascular and renal outcomes (reduced cardiovascular death and hospitalization for HF and slowed the decline in eGFR) in patients with HFrEF with or without diabetes
Böhm et al. [11]	2021	3730	RCT	To explore the interplay between HFrEF and systolic blood pressure (SBP)	Systolic blood pressure had no significant interaction with drug therapy. Empagliflozin significantly improved cardiovascular and renal outcomes (reduced cardiovascular death and hospitalization for HF and slowed the decline in eGFR) in patients with HFrEF across all SBP groups
Fitchett et al. [12]	2019	7020	RCT	To explore the effects of empagliflozin across three-point MACE, cardiovascular and all-cause death, and hospitalization for heart failure	Empagliflozin is beneficial in patients with T2DM and ASCVD irrespective of history of MI or stroke and across the spectrum of estimated cardiovascular risk
Lee et al. [13]	2021	105	RCT	To scrutinize the effects of SGLT2 inhibitors on cardiac structure and function in HFrEF	Empagliflozin significantly reduced (LVESVI/LVEDVI/N-terminal pro-B-type natriuretic peptide) in patients with HFrEF and T2DM or prediabetes
Omar et al. [14]	2020	70	RCT	To investigate the effects of empagliflozin on central hemodynamics (PCWP/CI ratio) in patients with HFrEF	Among patients with stable HFrEF, empagliflozin over 12 weeks reduced PCWP compared with the placebo group. There was no significant improvement in neither CI nor PCWP/CI at rest or exercise
Omar et al. [15]	2021	190	RCT	To investigate the outcome of SGLT2 inhibitor empagliflozin compared with placebo on cardiac remodeling and volumes in patients with HFrEF	Empagliflozin significantly reduced LVESVI/LVEDVI/LAVI/left ventricular (LV) mass index compared with placebo at 12-week follow-up with no change in ejection fraction
Santos-Gallego et al. [16]	2021	84	RCT	To explore the effect of empagliflozin on left ventricular function and volumes, functional capacity, and quality of life in non-diabetic HFrEF patients	Empagliflozin was associated with the significant reduction of LVEDV, LVESV, LV mass, and LV sphericity; improvements in LVEF; and significant improvement in peak oxygen consumption, oxygen uptake efficiency slope, six-minute walk test, and quality of life
Zannad et al. [17]	2020	3730	RCT	To investigate the effect of empagliflozin on cardiovascular and kidney outcomes across the spectrum of kidney function	Empagliflozin significantly improved cardiovascular and renal outcomes (reduced cardiovascular death and hospitalization for HF and slowed the decline in eGFR and reduced the risk of composite kidney outcome) in patients with HFrEF with and without CKD

Discussion

Effects of Empagliflozin on Cardiovascular and Renal Outcomes

Outcomes of empagliflozin across the diabetic and cardiovascular spectrum: Studies consistently show that SGLT2 inhibitors, as opposed to other antihyperglycemic medications, reduce the likelihood of heart failure hospitalizations and serious renal outcomes in diabetic patients [18-20]. These advantages for the heart and kidneys are unique to SGLT2 inhibitors [21]. Regardless of a person's glycosylated hemoglobin (HbA1c), investigations have shown that empagliflozin has an impact on both cardioprotective and nephroprotective effects [21]. Beta-blockers, renin-angiotensin system inhibitors, and background therapy with mineralocorticoid receptor antagonists and ARB/neprilysin inhibitors all have a synergistic impact [22]. Anker et al. reported that while the risk was equivalent in individuals who were prediabetic and normoglycemic, it was 40% greater in diabetic patients compared to non-diabetic patients for cardiovascular death, heart failure hospitalization, and unfavorable renal outcomes [10]. Empagliflozin significantly improved cardiovascular and renal outcomes in patients with heart failure with reduced ejection fraction irrespective of patients' baseline diabetic status and also across the continuum of HbA1c [10]. This shows that the level of glycemic control in a diabetic patient has no bearing on the effect of empagliflozin on cardiorenal advantages.

Ischemic cardiovascular disease manifests as a number of multifactorial disorders that worsen mostly as a result of atherothrombotic events, leading to myocardial infarction, heart failure, and death [12]. Patients with T2DM may be at a high risk for future cardiovascular events. Recent studies show that people with diabetes mellitus and cardiovascular disease are at a higher risk for hospitalization and mortality [12].

In HFrEF Patients Across the Systolic Blood Pressure Spectrum

In addition to helping people with heart failure, SGLT2 inhibitors also lower systolic blood pressure in people with diabetes and hypertension [23,24]. However, research was necessary to know if these drugs could produce hypotension as an adverse consequence [25,26]. Patients with lower systolic blood pressure were either not prescribed these drugs or only received minimal doses [25,26]. Lower systolic blood pressure was linked to a higher risk of one-year all-cause death and readmission rates for patients with heart failure, according to an observational study [27]. In their trial, Böhm et al. found that the overall number of heart failure hospitalizations and the relative risk reduction of the primary outcome by empagliflozin were equal across all SBP groups [11]. Empagliflozin decreased the probability of a composite renal outcome and attenuated the slope of eGFR reduction across all SBP categories [11]. These trials can be contrasted to highlight the fact that patients with low SBP and HFrEF tolerated the empagliflozin medication well, did not experience a drop in SBP, and did not see an increase in symptomatic hypotension rates [11].

In HFrEF Patients With Chronic Kidney Disease (CKD)

Heart failure patients frequently have concurrent CKD, and the likelihood of heart failure increases as kidney function declines [28]. On dialysis, 44% of patients have heart failure, and half of these had a decreased ejection fraction [28]. Angiotensin-converting enzyme inhibitor (ACEI) therapy is the first line of treatment for heart failure [29]. However, patients with severe chronic renal disease and lower eGFR rates who begin ACEI therapy are at a higher risk of developing hyperkalemia within the first year of starting the medication [30]. These medications also raised creatinine levels after starting treatment, and this was linked to unfavorable cardiorenal outcomes in a graded manner even below the suggested guideline cutoff point of 30% increase in creatinine [31]. SGLT2 inhibitors have been shown to lower the risk of heart failure hospitalization and cardiovascular death in individuals with type 2 diabetes mellitus. It slows the eGFR decline and the progression of CKD (including the requirement for dialysis and renal transplantation) [17]. The effects of empagliflozin on cardiovascular death, heart failure hospitalization, eGFR decrease, and composite kidney outcome were enhanced over a wide range of baseline kidney function, including patients with eGFR as low as 20 ml/minute/1.73 m^2^, according to Zannad et al.'s analysis of the EMPEROR-Reduced (empagliflozin outcome trial in patients with chronic heart failure with reduced ejection fraction) [17].

Effects of Empagliflozin on Cardiac Structure/Function and Hemodynamics in HFrEF Patients

To control and overcome the impaired central and peripheral metabolic situation in heart failure, a number of compensatory mechanisms are activated, including adaptive mechanisms on the cellular level and neurohormonal mechanisms (renin-angiotensin-aldosterone system and sympathetic-adrenergic system) [32]. In heart failure, the persistent neurohormonal stimulation alters cellular expression and structural cell interactions (fibrosis and hypertrophy), and it also serves as a predictor of death [32]. The disorganization of cardiomyocytes, ventricular hypertrophy (ventricular mass), chamber dilatation, and therefore increased wall tension and decreased subendocardial perfusion are structural alterations that may worsen heart function [32]. Myocardial contractility, preload (diastolic filling volume and maximal stretch length), and afterload (the resistance of the peripheral vasculature and aortic compliance) are all necessary for the left ventricle to operate [32]. The heart's capacity to alter its contractile force and hence increase stroke volume as a result of increased preload is known as the Frank-Starling mechanism [32]. It is reliant on the link between length (sarcolemma) and tension (force) at the cellular level [32]. Depending on the severity of the heart failure, this connection may change. It may also come to a standstill when the heart can no longer contract harder after being stretched more [32]. Stroke volume is depicted as pressure-volume loop and will be influenced by preload, afterload, and inotropy [32].

**Figure 2:**
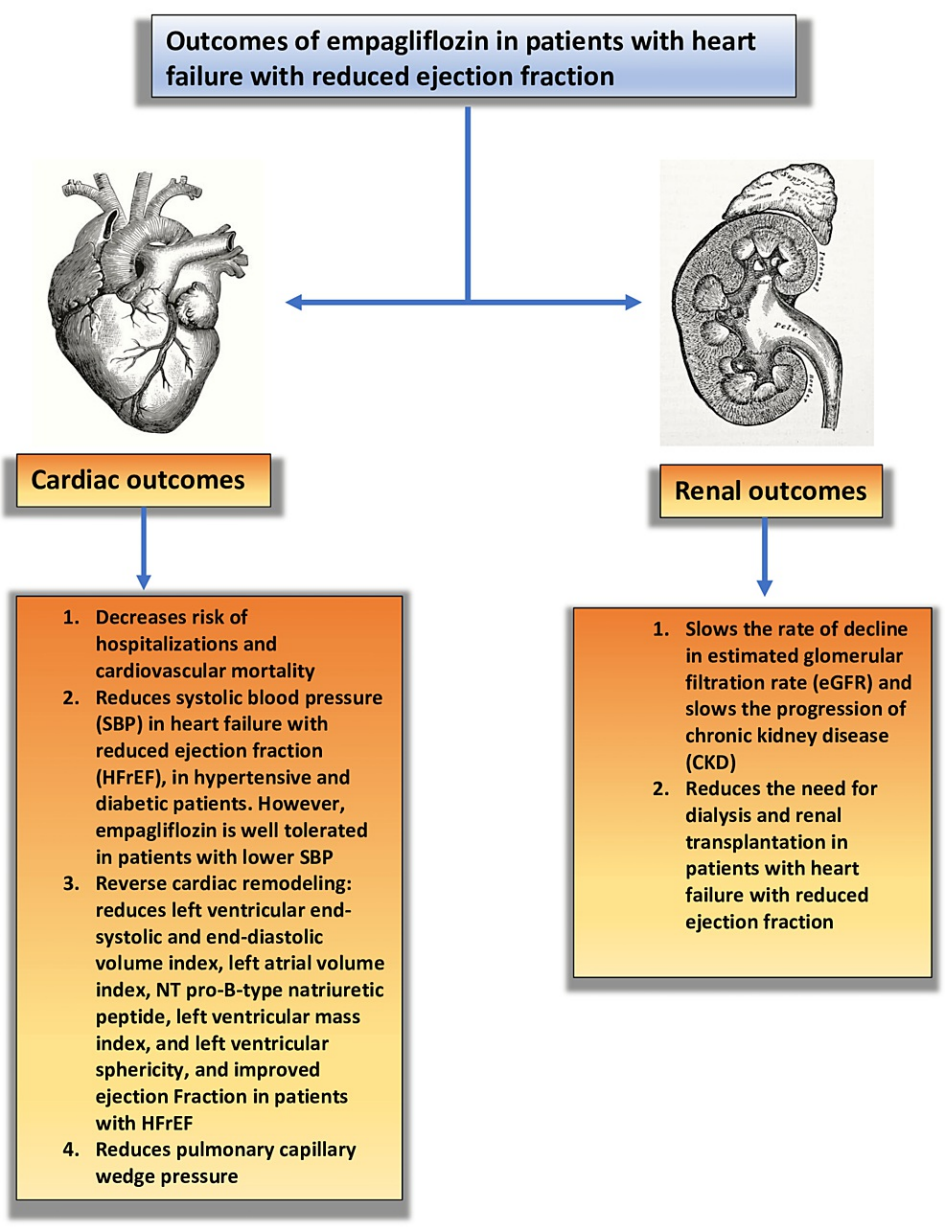
Summary of the cardiovascular and renal outcomes of empagliflozin in patients with heart failure with reduced ejection fraction Image credits: Sreekartthik Athiyaman NT: N-terminal

Clinical outcomes in HFrEF are better when using pharmacological or biological treatments that lessen or reverse cardiac remodeling [33]. The N-terminal pro-B-type natriuretic peptide (NT-pro-BNP) reduction is evidence that the SGLT2 inhibitors have a beneficial effect on cardiac remodeling [13]. Empagliflozin decreased left ventricular end-systolic volume index, left ventricular end-diastolic volume index, and NT pro-B-type natriuretic peptide in patients with heart failure with reduced ejection fraction and type 2 diabetes mellitus and prediabetes, according to a recent study by Lee et al. in 2021 [13]. In a 12-week research by Omar et al., empagliflozin decreased LVESVI, LVEDVI, LAVI, and left ventricular (LV) mass index compared to the placebo group in patients with HFrEF and an ejection fraction of less than 40% [15]. Empagliflozin was found to lower pulmonary capillary wedge pressure (PCWP) but had no effect on the ratio of PCWP and cardiac index (CI) at maximal activity in a subsequent trial by Omar et al. in patients with HFrEF and type 2 diabetes in 2020 [14]. Empagliflozin decreased LVESV, LVEDV, LV mass, and LV sphericity and improved LVEF, peak oxygen consumption, and oxygen uptake efficiency slope, as well as the quality of life, in a study by Santos-Gallego et al. in patients with heart failure with reduced ejection fraction (EF) of <40% [16]. These recent trials found that empagliflozin reduced heart failure hospitalization and mortality by improving LV reverse remodeling in patients with HFrEF. The overall cardiovascular and renal outcomes are summarized in Figure 2.

Limitations

Although empagliflozin exhibited positive benefits on the cardiorenal system, its effects on patients with advanced cardiorenal disease were not considered, including those with SBP of ≤100 mmHg, patients in New York Heart Association (NYHA) class IV, and those with symptomatic hypotension. Due to their post hoc character, two of the investigations had limitations. The patients' initial characteristics showed some heterogeneity. Treatment was only administered for 36 weeks in order to analyze the left ventricular remodeling; long-term consequences were not examined. There is a need for more investigation that considers the uniformity and long-term effects of the characteristics. Additionally, more research on how empagliflozin affects people with severe renal and cardiac disease will help us learn more about its potential future applications.

## Conclusions

This systematic review demonstrated a multitude of benefits of empagliflozin in patients with heart failure with reduced ejection fraction. The favorable effects were seen on both cardiovascular and renal outcomes. There were certain anticipatory outcomes consistent with previous studies. However, these studies also displayed the need for a further extensive study to know the exact mechanism of action for its benefits. They also needed further evaluation of patients with severe cardiac and renal diseases. The studies showed a reduction in cardiovascular death and hospitalization rates for patients with heart failure. There were positive effects on cardiac structure, volumes, and hemodynamics in patients with heart failure with reduced ejection fraction (HFrEF). This results in a reversal of left ventricular cardiac remodeling, which improves morbidity and overall mortality. This systematic review also showed that the effects of empagliflozin were unaffected by the varying systolic blood pressure, cardiovascular risk, and diabetic status.

There was an overall improvement in patients with chronic kidney disease, including patients on dialysis over time. This was correlated with the demonstration of the slowed decline of the estimated glomerular filtration rate (eGFR) slope. When it comes to using empagliflozin in HFrEF patients, the benefit outweighed the risk according to our study.
